# Higher-order structure of DNA determines its positioning in cell-size droplets under crowded conditions

**DOI:** 10.1371/journal.pone.0261736

**Published:** 2021-12-22

**Authors:** Takashi Nishio, Yuko Yoshikawa, Kenichi Yoshikawa

**Affiliations:** 1 Faculty of Life and Medical Sciences, Doshisha University, Kyoto, Japan; 2 Center for Integrative Medicine and Physics, Institute for Advanced Study, Kyoto University, Kyoto, Japan; Politechnika Slaska, POLAND

## Abstract

**Background:**

It is becoming clearer that living cells use water/water (w/w) phase separation to form membraneless organelles that exhibit various important biological functions. Currently, it is believed that the specific localization of biomacromolecules, including DNA, RNA and proteins in w/w microdroplets is closely related to their bio-activity. Despite the importance of this possible role of micro segregation, our understanding of the underlying physico-chemical mechanism is still unrefined. Further research to unveil the underlying mechanism of the localization of macromolecules in relation to their steric conformation in w/w microdroplets is needed.

**Principal findings:**

Single-DNA observation of genome-size DNA (T4 GT7 bacteriophage DNA; 166kbp) by fluorescence microscopy revealed that DNAs are spontaneously incorporated into w/w microdroplets generated in a binary aqueous polymer solution with polyethylene glycol (PEG) and dextran (DEX). Interestingly, DNAs with elongated coil and shrunken conformations exhibit Brownian fluctuation inside the droplet. On the other hand, tightly packed compact globules, as well as assemblies of multiple condensed DNAs, tend to be located near the interface in the droplet.

**Conclusion and significance:**

The specific localization of DNA molecules depending on their higher-order structure occurs in w/w microdroplet phase-separation solution under a binary aqueous polymer solution. Such an aqueous solution with polymers mimics the crowded conditions in living cells, where aqueous macromolecules exist at a level of 30–40 weight %. The specific positioning of DNA depending on its higher-order structure in w/w microdroplets is expected to provide novel insights into the mechanism and function of membraneless organelles and micro-segregated particles in living cells.

## Introduction

Living organisms on Earth maintain their lives by adopting a macromolecular crowded cellular environment, i.e., cytoplasmic solution contains 30–40 weight % of macromolecules, including skeletal proteins, RNA, DNA, etc. [1–4]. It has been well established in polymer physics that polymer chains at a relatively high concentration in good solvent conditions exhibit a so-called depletion effect [5–11] due to the entropic contribution of their elongated conformation. In an aqueous solution of a mixture of semiflexible and flexible polymers, similar to the usual solution conditions for cytoplasm, semiflexible polymers are depleted into a folded compact state or a segregated state by self-assembly [12–14]. In relation to the segregation phenomenon, or w/w phase separation in aqueous solution with multiple macromolecules, membraneless organelles, such as nucleolus, ribosomes, P-body, and stress granules, have recently been attracting considerable interest in the biological sciences [15–17]. The stability of these membraneless organelles, together with experimental studies to construct their models [17, 18], have frequently been interpreted in terms of liquid-liquid phase separation [19, 20]. As for the stability and function of membraneless organelles, it is becoming clear that intrinsically disordered proteins (IDPs) exhibiting flexible domains play important roles in a wide variety of cellular functions [17, 21, 22]. Such experimental observations suggest that research on the depletion effect in a cytoplasmic environment under crowded macromolecular conditions would promote the basic understanding of living systems. In relation to this, it is noted that not a small number of studies of selective partition of various chemical compounds have been carried out in terms of ATPS, Aqueous Two Phase System [23–25]. We have recently been studying the physico-chemical properties of micro-phase separation in binary hydrophilic polymer solution, with flexible linear polymer chains and stiff branched polymer chains; for these polymers, we have adopted polyethylene glycol (PEG) and dextran (DEX), respectively, as a simple model of a crowded macromolecular environment in living systems [26–32]. Interestingly, it has been shown that, when double-strand DNA and actin filament are mixed in a binary polymer solution of PEG/DEX, these biological macromolecules selectively accumulate in DEX-rich droplets under micro w/w phase separation. It was reported that RNA replication is promoted in DEX-rich droplet generated through micro separation of PEG/DEX solution, where the replication machinery is concentrated [33]. Theoretical studies have also suggested the specific localization of semiflexible polymer depending on the degree of crowding in a micro-confinement as in living cells [34, 35]. As an extension of these studies, in the present article we have performed the research to clarify how genome-size DNA (T4 GT7 bacteriophage DNA; 166kbp) behaves under cell-sized w/w microphase separation by use of single-DNA observation under the control of its higher-order structure with different concentrations of spermidine, SPD, as one of the representable biological polyamines. Currently, not a small number of studies have been reported on DNA condensation caused by polyamines and other multivalent cations, reveling that polyamines induce compaction/condensation of DNA and their potencies for inducing DNA compaction are sensitively dependent on their valency and structural geometry/isomerization [36–38]. Recently, it was found that, through in vitro experiments with cell-free gene expression assay, polyamines promote gene expression just below the concentration to induce tight compaction but inhibit gene expression completely at higher concentrations [39–42]. It was revealed that such biphasic effect of polyamines neatly concerns with transition of higher order structure of DNA molecules [39, 41–44]. It would be highly expected that such characteristic function of polyamine on the structure and function of DNA play an important role in living cellular media accompanied with microphase separation. Thus, in the present study, we have carried out the single DNA observation in the solution environment under water/water microphase separation for the DNA molecules with different higher-order structures at different concentrations of SPD.

## Materials and methods

### Materials

Spermidine trihydrochloride (SPD) was purchased from Nacalai Tesque (Kyoto Japan). Polyethylene Glycol 6,000 (PEG), Dextran 200,000 (DEX) and the antioxidant 2-mercaptoethanol (2-ME) were purchased from FUJIFILM Wako Pure Chemical Corporation (Osaka, Japan). T4 GT7 bacteriophage DNA (166 kbp with a contour length of 57 μm) and Tris-hydrochloride acid buffer (ph7.5) were purchased from Nippon Gene (Tokyo, Japan). The dimeric cyanine fluorescent dye YOYO-1 (1,10-(4,4,8,8-tetramethyl-4,8-diazaundecamethylene)bis[4-[(3-methylbenzo-1,3-oxazol-2-yl)methylidene]-l,4-dihydroquinolinium] tetraiodide) was obtained from Molecular Probes Inc. (OR, USA).

### Methods

#### Preparation of w/w microdroplets entrapping DNAs

**Fig 1:**
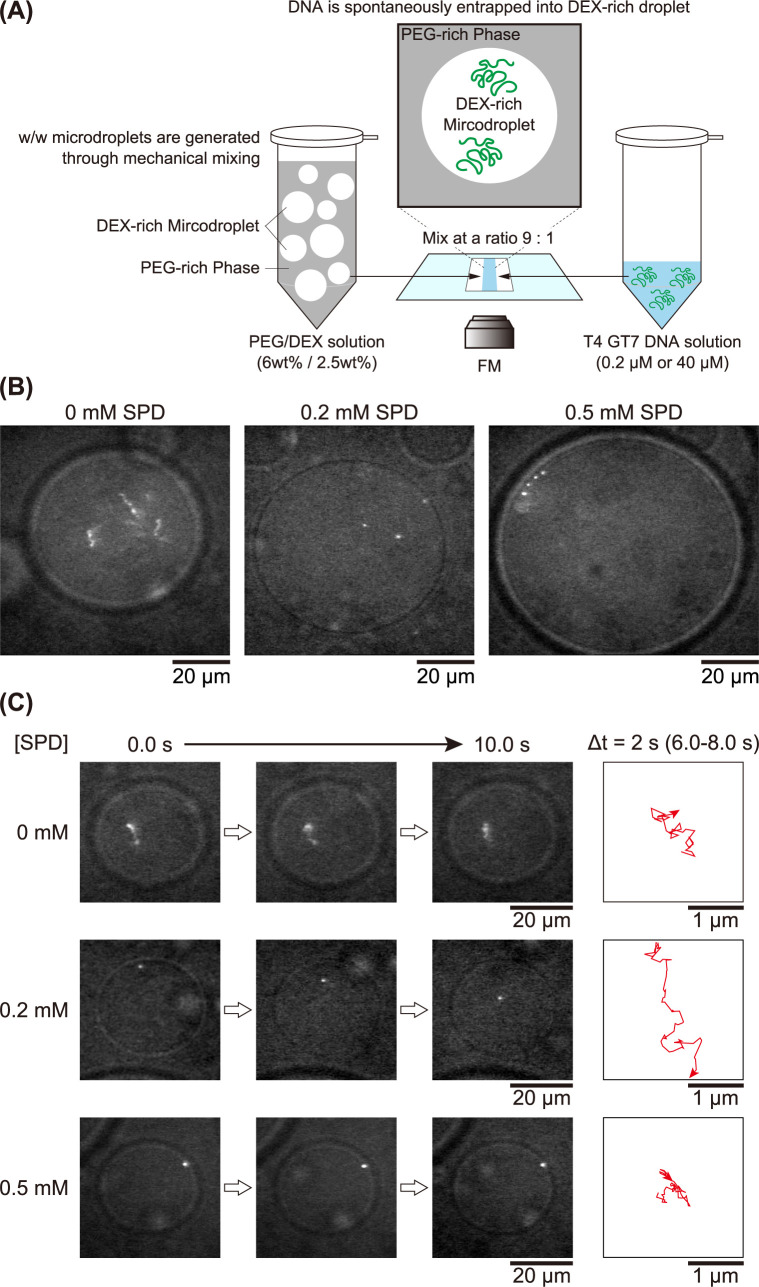
FM observations of w/w microdroplets entrapping single T4 GT7 DNA molecules. The microdroplets are generated through phase separation of PEG/DEX (6 and 2.5 wt%) solution with a low DNA concentration (0.2 μM in nucleotide units) at different concentrations of SPD. The inner and outer solutions of the droplet are DEX-rich and PEG-rich, respectively. (A) Schematic representation of the experimental procedure to prepare the PEG/DEX micro-segregated solution in the presence of DNA. (B) Multiple T4 GT7 DNA molecules in w/w microdroplets at different concentrations of SPD. (C) Time-successive images are shown on the left side. Single T4 GT7 DNA molecules in w/w microdroplets exhibiting Brownian motion. The trajectories of DNA molecules for the period of Δt = 2 s between t = 6 s and t = 8 s are shown in right side.

To prepare w/w microdroplets, we adopted an aqueous two-phase system (ATPS) by using 6 weight % PEG and 2.5 weight % DEX. As has been reported, this composition corresponds to a phase-segregated state near a bimodal line in the phase-diagram [26]. After mechanical mixing of this PEG/DEX solution, the solution becomes cloudy accompanied by the generation of w/w microdroplets and then gradually fuse each other toward macro-scale phase separation over several hours. To prepare the PEG/DEX micro-segregated solution in the presence of DNA, we mechanically agitated the PEG/DEX solution by a vortex mixer and then mixed it with the T4 GT7 DNA solution at a ratio of 9:1 on a glass slide (See the schematic representation in Fig 1A). FM observations were conducted at DNA concentrations of 0.2 μM and 40 μM (i.e., the DNA concentration in the DNA solution was fixed at 2 μM and 400 μM in nucleotide units) with the addition of YOYO-1 (0.05 μM and 0.5μM, respectively). The final concentrations of other compounds were 10 mM Tris-HCl buffer solution at pH 7.5, 4% (v/v) 2-ME and the desired concentrations of SPD.

### Fluorescence microscopy (FM) observation

We performed FM observations with an inverted fluorescence microscope (Axiovert 200, Carl Zeiss, Oberkochen, Germany) equipped with a 100× oil-immersion objective lens. Fluorescent illumination was performed using a mercury lamp (100 W) with a filter set (Zeiss-10, excitation BP 450–490; beam splitter FT 510; emission BP 515–565). To visualize both individual DNA molecules and contours of w/w microdroplets simultaneously, we performed the observation under constant transmitted light. We recorded the images onto a DVD at 30 frames per second through a high-sensitivity Electron Bombarded Charge-Coupled Device (EBCCD) camera (Hamamatsu Photonics, Shizuoka, Japan). We analyzed images with the image-processing software ImageJ (National Institute of Mental Health, MD, USA).

## Results and discussion

### Spontaneous entrapment of DNA into droplets

Fig 1B exemplifies FM images of single T4 GT7 DNA molecules in w/w microdroplets at each concentration of SPD. As shown in the left panel, without the addition of SPD, DNA molecules exhibit an elongated random coil conformation inside the DEX-rich droplet. This spontaneous entrapment of DNA corresponds to our past observations that double-stranded DNA accumulates in DEX-rich w/w microdroplets, where visualization of individual DNA molecules was not possible [26, 29]. In the presence of 0.2 mM SPD, coexistence [37] of the elongated coil and dot-like globule states inside DEX-rich droplets is observed, where many of the DNAs have a globule conformation and only a small fraction exhibit a coil conformation. During the observation period of several secs to several tens of secs, we confirmed that the globule DNA molecules exhibit Brownian motion inside DEX-rich droplets; sometimes the globule DNA molecules make contact with the droplet interface for several secs and return back to the interior under fluctuating motion. In the presence of 0.5 mM SPD, all the DNA molecules exhibit a globule conformation. Notably, all of the globule DNA molecules tend to localize at the interface in microdroplets. Based on these observations, we analyzed the time-trace of the fluctuating motion of individual DNA molecules at different concentrations of SPD. Fig 1C shows the time trajectories of the center of mass of single DNA molecules. In the absence of SPD, elongated coil DNA molecules exhibit rather slow fluctuations for both intrachain and translational Brownian motion. In contrast, in the presence of 0.2 mM SPD, the globule DNA exhibits much greater translational fluctuation compared to that in the coil state. In the presence of 0.5 mM SPD, the globule DNA molecule is situated at the droplet interface without almost no apparent translational or intrachain Brownian motion.

### Evaluation of the actual size of entrapped DNAs through an analysis of Brownian motion

As mentioned above, it is becoming clear that DNA molecules exhibit specific positioning in DEX-rich droplets, depending on the conformation of the DNA molecules. Regardless of this useful observation, FM images of freely moving DNA molecules are limited by a relatively low resolution, compared to the usual optical microscopic observation for specimens absorbed/fixed on the surface of slide glass. It is known that the actual resolution limit is on the order of 1 μm for individual single DNA molecules [45, 46]. To obtain information on the actual size of DNA in DEX-rich droplets, we analyzed its Brownian motion by tracking the time-dependent motion of individual molecules and evaluated the hydrodynamic radius *R*_H_ of DNA. (The details of method of the analysis of the Brownian motion are shown in S1 File and S1 Fig)

First, we estimated *η*_md_, the viscosity of the internal solution of w/w microdroplets, by using monodisperse polystyrene microspheres (2.5% Solids-Latex), 1.00 μm from Polysciences Inc (Warrington, PA, USA). The *R*_H_ of microbeads was 0.47 ± 0.06 μm in pure water. The viscosity of pure water *η*_aq_ is 0.89 mPa・s at 298 K. Next, we measured the diffusion constant *D* of polystyrene beads entrapped within the DEX-rich droplet under the same solution conditions for entrapped DNA molecules as in Fig 1. From these measurements, we evaluated the viscosity within the DEX-rich droplets.; *η*_md_ = 4.2 ± 0.3 mPa・s at 298 K. *R*_H_ of microbeads and *η*_md_ were evaluated from the statistical treatment of the data points with more than one hundred for each time-period from the analysis of the time-dependent traces for five single-microbeads (see S1 File).

The above experimental and analytical procedure clarified that, at SPD = 0 mM, DNA molecules entrapped in a DEX-rich droplet exhibit an elongated coil conformation with *R*_H_ = 1.03 ± 0.11 μm. At 0.2 mM SPD, the DNA within a DEX-rich droplet exhibits a shrunken state with 0.31 ± 0.05 μm. Each *R*_H_ was evaluated from the statistical analysis on the time-dependent traces of the translational fluctuation of individual DNA with more than five molecules (As for the detail of the statistical treatment, see S1 File). This difference in *R*_H_ indicates that the effective volume changes on the order of (0.311.03)3≈0.03, which accompanies the change from SPD = 0 mM to SPD = 0.2 mM. In our past study to measure the Brownian motion of single DNA molecules, we reported that the hydrodynamic radius of the tightly packed globule state of T4 GT7 DNA is *R*_H_ = 0.06 ± 0.1 μm [47]. This suggests that the volume change caused by the transition from an elongated coil to a compact globule is on the order of (0.061.03)3≈0.0002, and the shrunken state of DNA observed at 0.2 mM has a larger effective volume than the compact globule state. The globule state exemplified at SPD 0.5 mM in Fig 1 is attributable to the tightly compact state, although free Brownian motion was not observed due to adsorption onto the droplet interface. In relation to the shrunken state of DNA, it has been reported that, for the coil-globule transition of single DNA molecules induced by polyamines, a flower-like conformation appears just below the threshold concentration to induce the compact globule [39–42]. The difference in physico-chemical properties between the shrunken state and compact state of DNA has also been recently revealed experimentally [48]. Interestingly, for experiments on *in vitro* gene expression, the promotion and inhibition of gene expression are observed for the shrunken state and compact globule states, respectively [39, 41–44].

### Droplets with a higher concentration of entrapped DNA

**Fig 2:**
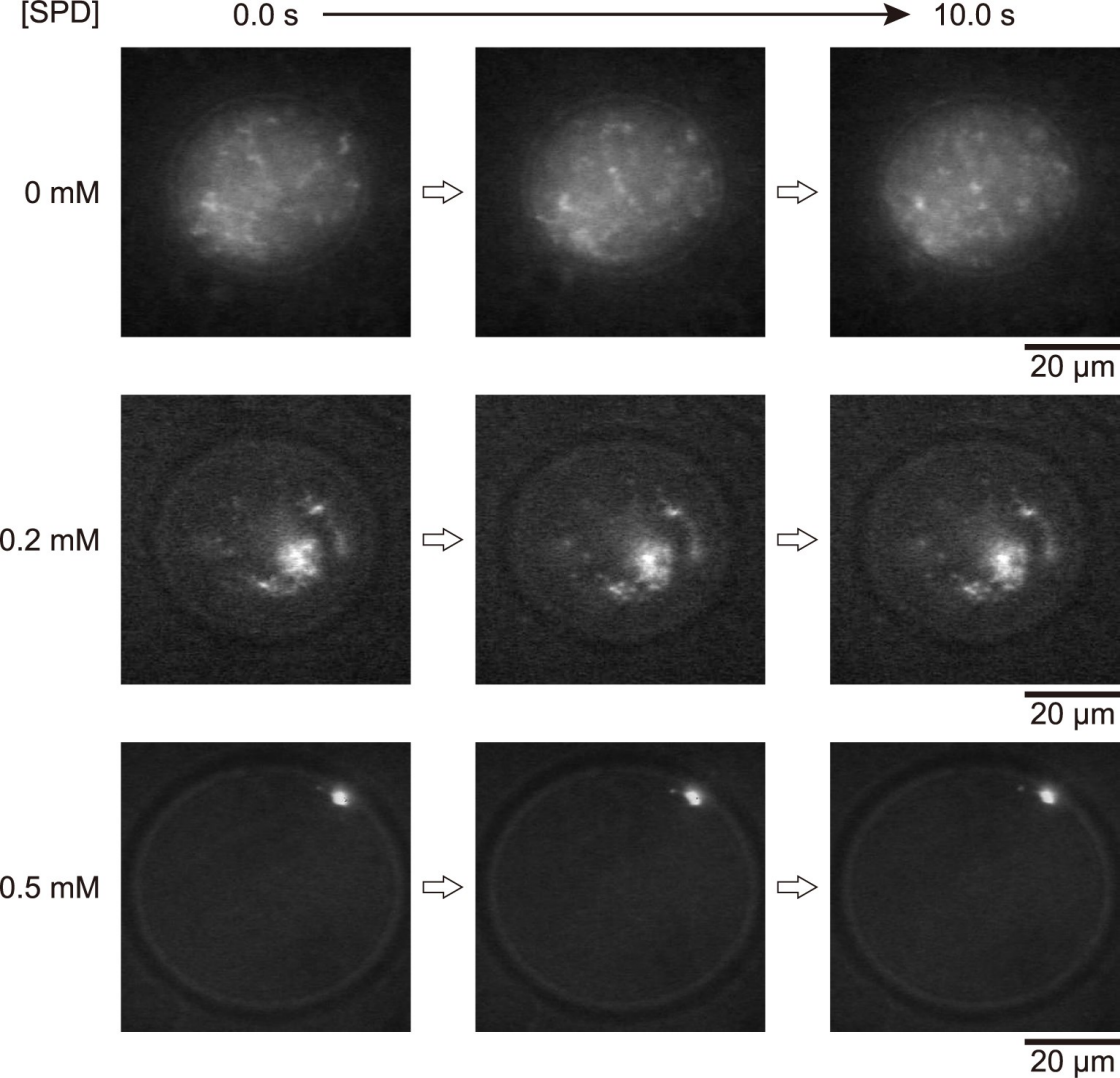
FM observation of w/w microdroplets entrapping several T4 GT7 DNA molecules. The microdroplets were generated through phase separation of PEG/DEX solution under the conditions similar to those in Fig 1, except for the DNA concentration of 40 μM in nucleotide units. The total observation time is 10 s.

To further investigate the nature of the localization of DNA in w/w microdroplets, we performed additional FM observations at higher DNA concentrations. Fig 2 shows typical real-time imaging of T4 GT7 DNA molecules in microdroplets at 0, 0.2 and 0.5 mM SPD. The final DNA concentration was fixed at 40 μM, which is 200 times higher than that in the single-molecule observation shown in Fig 1. In the absence of SPD, DNA molecules with an elongated coil conformation are widely distributed in microdroplets. In the presence of 0.2 mM SPD, DNA molecules tend to assemble with each other, but are still widely distributed within the droplet. On the other hand, in the presence of 0.5 mM SPD, DNA molecules aggregate in a condensed manner by forming a single optical spot in FM images. The compact DNA aggregate is situated at the interface of the droplet in a stationary manner, being similar to the positioning of single compact DNA in Fig 1 (SPD 0.5 mM).

### Real-time observation of the folding transition of a single DNA inside a droplet

**Fig 3:**
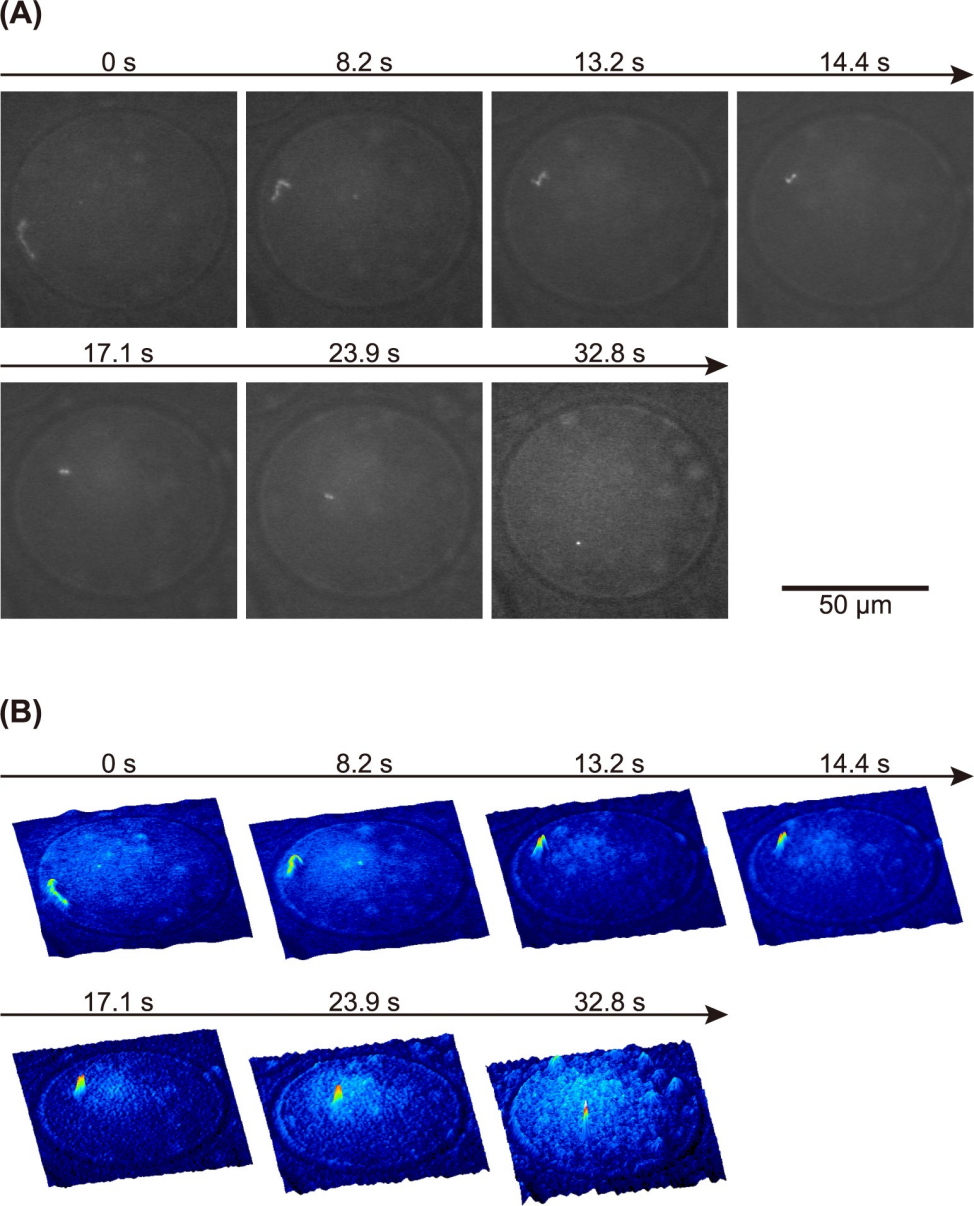
Real-time observation of the folding transition of a single T4 GT7 DNA molecule in a w/w microdroplet. (A) FM images reveal that, under thermal fluctuation, the conformation of a single DNA changes from an elongated coil to a compact globule. The solution composition was similar to that in Fig 1 in the presence of 0.2 mM SPD. The folding transition was generated spontaneously during FM observation of the droplet. (B) Corresponding quasi-3D profiles colored with the fluorescence intensity for the images in (A).

Fig 3 exemplifies the real-time imaging of the higher-order structural transition of T4 GT7 DNA within a w/w microdroplet. In the presence of 0.2 mM SPD, a DNA molecule gradually changed its conformation from an elongated coil to a shrunken state on a time-scale of 20–30 sec. During Brownian motion in the coil state, two bright spots appear spontaneously at a point on the chain and the apparent contour length of the chain slowly decreases. Finally, a single bright spot appears, while maintaining translational fluctuation as in Fig 1C at 0.2 mM SPD. A similar experimental observation of the spontaneous transition from an elongated coil to a compact globule, as a kind of nucleation-growth kinetics, caused by the depletion effect in a crowded polymer solution has been reported [49]. As far as we are aware, this is the first report on the direct observation on the kinetic process of folding transition into globule state caused by multivalent cation.

### Working hypothesis on the mechanism and biological significance of the specific localization of DNA depending on its higher-order structure

Here, we discuss the mechanism of the specific localization of DNA molecules depending on their higher-order structure. The underlying mechanism of the spontaneous entrapment of DNA has been discussed in relation to the mechanism of w/w phase separation with a binary polymer solution, such as in a PEG/DEX system [26, 29, 50]. The manner of packing of crowded polymers is quite different between PEG-rich and DEX-rich solutions. In the DEX-rich phase, a nanosized void-space exists due to the stiff backbone and branched conformation of DEX, whereas the PEG-rich phase is fully occupied with flexible chains with a random conformation. For double-strand DNA with a diameter of 2 nm, the persistence length is around 170 bp (ca. 50 nm). Thus, T4 GT7 DNA behaves as a semiflexible polymer chain. It has been revealed that such semiflexible DNA molecules are folded into a compact/ condenses state and/or are aligned in a liquid-crystalline-like phase in the presence of a crowded flexible polymer such as PEG, through the effect of so-called depletion interaction [12, 13, 51]. For the phase-separated PEG/DEX solution, semiflexible DNA is depleted from PEG-rich phase due to the crowding effect as the flexible polymer. Inside the DEX-rich phase, elongated DNA is allowed to exhibit thermal fluctuation in void micro-space existing owe to branched-structures of DEX, which causes the preference positioning of DNA inside the DEX-rich droplet.

On the other hand, tightly folded compact DNA behaves like a colloidal particle on a scale of 0.1μm, suggesting that such compact DNA cannot enter into the nanosized void-space inside the DEX-rich droplet, and is depleted from the PEG-rich phase. As a result, compact globule DNA as well as assemblies of multiple compact DNAs should be localized at the interface between the PEG-rich and DEX-rich solutions, which is caused by the depletion effects from both phases. It is noted that such size effect is essentially important in depletion effect, as has been demonstrated in our recent study [26, 29]. That is, short fragment DNA equally distributes for the both phases of PEG/DEX, whereas giant DNA is selectively partitioned into the DEX-rich phase.

The above-mentioned observations on the specific localization either inside or boundary of the droplet, depending on the conformation of giant DNA molecule is expected to stimulate further studies on the function of micro phase-segregation in living cellular systems [15–22]. Recently, we have shown that red-blood cells and epithelial cells are spontaneously entrapped into DEX-rich droplets under the condition of phase-separation with DEX/PEG aqueous solution [52]. Interestingly, the manner of preferential localization, inside or boundary of the droplets, is controlled by changing the relative composition of DEX and PEG. Such experimental trend suggests that further experimental trials by changing the composition of DEX and PEG, including the replacement of the aqueous macromolecules by other bio- and synthetic-polymers [23, 53], would be promising. In relation to the manner of partition of macromolecules in w/w phase separation, it is getting clear that selectivity of the entrapment is highly dependent on the size and length of biopolymers including DNA and actin [29].

In relation to the specific localization of DNA dependent on its higher-order structure as observed in the present study, recent numerical studies with a Monte Carlo simulation have reported that a rigid particle tends to localize at the periphery of a confinement under crowded conditions, and that this localization switches to the interior for a soft particle [54, 55]. This preference of the soft particle for the boundary corresponds to our observation, i.e., DNA with an elongated coil conformation tends to accumulate at the interior of the droplet as observed in the present study.

## Conclusion

In summary, 1) DNA molecules were spontaneously entrapped into DEX-rich droplets in phase-separated PEG/DEX solution. 2) The entrapment of DNA is attributable to the different physico-chemical properties between PEG and DEX, which are flexible and branched rigid polymer skeletons, respectively. 3) The conformational transition of the higher-order structure of DNA entrapped in the droplet was caused by the addition of a cationic polyamine, SPD. 4) The localization of single DNAs was critically dependent on their conformation. DNAs with elongated coil and shrunken conformations exhibit Brownian fluctuation inside the droplet, whereas tightly packed compact globules adhere to the droplet interface in a stationary manner. 5) Such specific localization of DNA depending on its conformation was also observed for assemblies of many DNA molecules. 6) The transition from an elongated coil into a shrunken state is a rather slow process that occurs on the order of several tens of seconds. The present results may lead to new perspectives on the actual role of w/w phase separation in the formation of organelles. As related subjects, it was recently found that the boundary constraints of the nucleolus and nuclear envelope play essential functions through micro-phase separation with multiple biomacromolecules [56, 57] and that active and repressed chromatin separates from the nuclear interier and form a peripheral layer as obseved through live imaging [58, 59].

## Supporting information

S1 File Method of evaluation on the hydrodynamic radius *R*_H_ of DNA. (PDF)

S1 Fig Evaluation of the hydrodynamic radius *R*_H_ of T4 GT7 DNA molecules from an analysis of Brownian motion. (PDF)
