# Supplementary material for: Higher-order structure of DNA determines its positioning in cell-size droplets under crowded conditions
Source: PLoS One. 2021 Dec 22;16(12):e0261736. doi: 10.1371/journal.pone.0261736 (PMC8694483; doi:10.1371/journal.pone.0261736)
Supplement: S1 File — (PDF) [file pone.0261736.s001.pdf]

Supporting information

S1 File. Method of evaluation on the hydrodynamic radius R_H of DNA.

To gain further insight on the actual conformation of DNA molecules by overcoming the problem of the relatively low resolution with the FM observation, we analyzed the Brownian motion for the translational freedom of individual DNA molecules and evaluated the diffusion constant *D* of individual DNA molecules. We adopted the method of analysis of the Brownian motion by following the procedure in past studies [S1-S7]. We measured the time-dependent change of the center of mass of single DNA molecules for the duration of 10 sec. For the evaluation of average value of square displacement, we adopted the time-window for 3 sec, by taking the starting time points t_0 of the random walk at each 1 second for the DNA molecules more than 5 specimens. This means that each data point shown in S1 Fig. is the average value for more than 100 raw values of the displacement. As exemplified in S1 Fig, the plot of the mean square displacement vs. Δt (= $t - t_0$) tends to deviate toward the larger value. We have made it clear that, through the studies reported in the literatures [S1-S7], such deviation from the linear relationship is attributed to the existence of a small but nonnegligible convectional flow. Since the rate and direction of the convectional flow were almost during the observation period (on the order of several tens seconds) for the individual measurements,

we eliminated the effect of convective flow by adopting the relationship as in Eq (1) [S1-S7]:

$$\langle(\boldsymbol{r}(t)-\boldsymbol{r}(0))^2\rangle = 4Dt + A(t-t_0)^2, \qquad (1)$$

where $\boldsymbol{r}(t) = (r_x, r_y)$ is the position of the center of mass for a DNA. $\langle(\boldsymbol{r}(t)-\boldsymbol{r}(0))^2\rangle$ is the mean-square displacement and A is a constant related to the convectional flow. The effective hydrodynamic radius R_{H} of a single DNA molecule was evaluated base on the Stokes-Einstein relationship given in Eq (2) [S8, S9]:

$$R_H = \frac{k_B T}{6\pi\eta D} \qquad (2)$$

where k_{B} is the Boltzmann constant, η is the viscosity of the solvent, and T = 298 K. R_{H} of microbeads and η_{md} were evaluated from the statistical treatment of the data points with more than one hundred for each time-period from the analysis of the time-dependent traces for five single-microbeads, being similarly to the method of the estimation on R_{H} on single DNA molecules.

It is noted that the original movies are available from the following repository: 10.5281/zenodo.5733908.

References

S1. Matsumoto M, Sakaguchi T, Kimura H, Doi M, Minagawa K, Matsuzawa Y, et al. Direct observation of brownian motion of macromolecules by fluorescence microscope. J Polym Sci, Part B:

Polym Phys. 1992;30(7):779-783.
S2. Yoshikawa Y, Yoshikawa K, Kanbe T. Daunomycin unfolds compactly packed DNA. Biophys Chem. 1996;61(2-3):93-100.
S3. Sato YT, Hamada T, Kubo K, Yamada A, Kishida T, Mazda O, et al. Folding transition into a loosely collapsed state in plasmid DNA as revealed by single-molecule observation. FEBS Lett. 2005;579(14):3095-3099.
S4. Araki S, Nakai T, Hizume K, Takeyasu K, Yoshikawa K. Hydrodynamic radius of circular DNA is larger than that of linear DNA. Chem Phys Lett. 2006;418(1-3):255-259.
S5. Chen N, Zinchenko AA, Yamazaki Y, Yoshikawa Y, Murata S, Yoshikawa K. Quantum dot probes for observation of single molecule DNA and a synthetic polyelectrolyte higher-order structure. Soft matter. 2010;6(12):2834-2841.
S6. Sato YT, Watanabe S, Kenmotsu T, Ichikawa M, Yoshikawa Y, Teramoto J, et al. Structural change of DNA induced by nucleoid proteins: growth phase-specific Fis and stationary phase-specific Dps. Biophys J. 2013;105(4):1037-1044.
S7. Ma Y, Yoshikawa Y, Oana H, Yoshikawa K. Marked Difference in the Conformational Transition of DNA Caused by Propanol Isomer. Polymers. 2020;12(7):1607.
S8. De Gennes P-G. Scaling Concepts in Polymer Physics. Ithaca, N.Y.: Cornell University Press; 1979.
S9. Doi M, Edwards SF. The theory of polymer dynamics: Oxford University Press; 1988.
