# Supplementary material for: Higher-order structure of DNA determines its positioning in cell-size droplets under crowded conditions
Source: PLoS One. 2021 Dec 22;16(12):e0261736. doi: 10.1371/journal.pone.0261736 (PMC8694483; doi:10.1371/journal.pone.0261736)
Supplement: S1 Fig — (PDF) [file pone.0261736.s002.pdf]

Supporting information

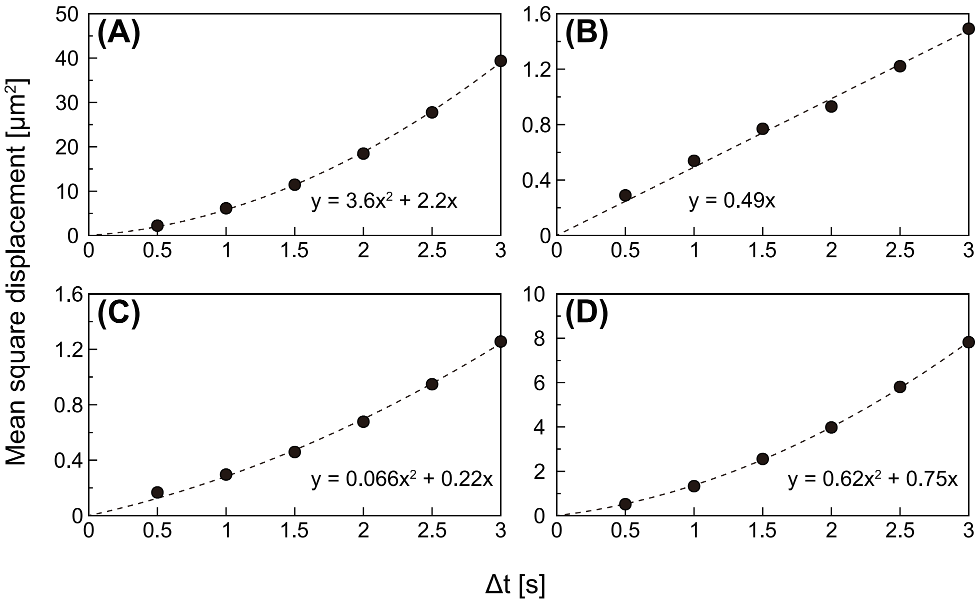

S1 Fig Evaluation of the hydrodynamic radius R_H of T4 GT7 DNA molecules from the analysis of Brownian motion.

Plot of the mean square displacement, $\langle (r(t) - r(0))^2 \rangle$, of polystyrene microbeads and DNA molecules as a function of time, based on real-time FM observations. The fitting curves were based on Eq (1) as in the main text. Mean square displacement of polystyrene microbeads: (A) in pure water; (B) in w/w microdroplets. Mean square displacement of single T4 GT7 DNA molecules in w/w microdroplets: (C) in the absence of polyamine; (D) in the presence of 0.2 mM SPD.
